# Effects of Swimming Exercise on Limbic and Motor Cortex Neurogenesis in the Kainate-Lesion Model of Temporal Lobe Epilepsy

**DOI:** 10.1155/2016/3915767

**Published:** 2016-05-22

**Authors:** Vasavi R. Gorantla, Amulya Sirigiri, Yulia A. Volkova, Richard M. Millis

**Affiliations:** ^1^Department of Anatomy, American University of Antigua College of Medicine, St. John's, Antigua and Barbuda; ^2^Department of Anatomy, Melaka Manipal Medical College, Manipal University, Manipal 576104, India; ^3^Gandhi Medical College, Hyderabad 500003, India; ^4^Department of Clinical Medicine, American University of Antigua College of Medicine, St. John's, Antigua and Barbuda; ^5^Department of Medical Physiology, American University of Antigua College of Medicine, St. John's, Antigua and Barbuda

## Abstract

Temporal lobe epilepsy (TLE) is a common neurological disease and antiseizure medication is often inadequate for preventing apoptotic cell death. Aerobic swimming exercise (EX) augments neurogenesis in rats when initiated immediately in the postictal period. This study tests the hypothesis that aerobic exercise also augments neurogenesis over the long term. Male Wistar rats (age of 4 months) were subjected to chemical lesioning using KA and to an EX intervention consisting of a 30 d period of daily swimming for 15 min, in one experiment immediately after KA lesioning (immediate exposure) and in a second experiment after a 60 d period of normal activity (delayed exposure). Morphometric counting of neuron numbers (NN) and dendritic branch points and intersections (DDBPI) was performed in the CA1, CA3, and dentate regions of hippocampus, in basolateral nucleus of amygdala, and in several areas of motor cortex. EX increased NN and DDBPI in the normal control and the KA-lesioned rats in all four limbic and motor cortex areas studied, after both immediate and 60 d delayed exposures to exercise. These findings suggest that, after temporal lobe epileptic seizures in rats, swimming exercise may improve neural plasticity in areas of the brain involved with emotional regulation and motor coordination, even if the exercise treatment is delayed.

## 1. Introduction

Temporal lobe epilepsy is associated with oxidative stress and apoptotic cell death in brain tissues and is often resistant to treatment with antiepileptic drugs, and progression of the disease produces adverse neurobehavioral sequelae [1, 2]. The CA1, CA3, and dentate gyrus are regions of the mammalian hippocampus which are known to be exquisitely sensitive to experimentally induced kainic acid seizures in rats, evidenced by the presence of ionotropic glutamate receptors with specific binding properties for kainate (kainate receptors) [3]. The hippocampus is also the most active site of neurogenesis in the mammalian brain [4]. Temporal lobe epileptic seizures may spread to the amygdala where apnea is shown to occur by virtue of connectivity between amygdala and brainstem respiratory network nuclei [5]. Amygdaloid expression of the proconvulsant neuropeptide CRH and robust connectivity between hippocampus and amygdala suggest that the amygdala should be affected by kainate-induced seizures similarly as the hippocampus [6]. Neurogenesis in the amygdala has not been extensively studied in that regard. Connectivity between these limbic areas and motor cortex is less direct and not very robust, likely relayed through the cingulate and insular cortices [7]. Motor deficits in temporal lobe epilepsy are uncommonly reported and the effects of kainate-induced seizures on motor cortex have not been systematically studied. Aerobic exercise is a treatment for inhibiting neuronal apoptosis associated with aging and maintaining neuronal populations and brain volume [8, 9]; however, whether delays in initiating aerobic exercise decrease its potential for efficacious neurogenesis following seizures is not known. Aerobic exercise augments neurogenesis in both humans and rats [10] but humans affected by temporal lobe epilepsy are often unable to maintain vigorous exercise regimens, without breaks, over the long term. The present study was, therefore, designed to test the hypothesis in rats that swimming exercise, begun in the immediate postictal period following kainic acid-induced chemical lesioning and seizures, is more effective as a stimulus for neurogenesis in the hippocampus, amygdala, and motor cortex than such exercise initiated after a 60 d delay.

## 2. Methods

This research was approved by the Ethical Clearance Committee of Manipal University.

### 2.1. Animals

Male Wistar rats (4-month-old) were used. All the cages were maintained in 12-hour light and 12-hour dark cycle in well-ventilated rooms within the Manipal University Animal House. All rats were fed* ad libitum* with a balanced diet containing 21.96% crude oil, 3.10% crude fiber, 7.37% ash, and 1.38% sand silica.

### 2.2. Experimental Design

Rats were divided into five groups: (1) normal control, (2) normal control + exposure to exercise, (3) sham control + exposure to exercise, (4) kainic acid-lesioned, and (5) kainic acid-lesioned + exposure to exercise. Rats in the normal control group remained undisturbed in the home cage. Rats in the normal control + exercise group were subjected to swimming exercise for 15 min/d. Rats in the sham control group were subjected to sham surgery. The sham surgery consisted of positioning the rats fixed in a stereotaxic apparatus. Burr holes were drilled in the skull using appropriate coordinates. A Hamilton syringe was lowered into the lateral ventricles bilaterally and removed. The scalp wounds were sutured and animals were replaced back in their home cage and were subjected to exercise. Rats in the lesion only group were given kainic acid (KA) bilaterally into the hippocampus using a Hamilton syringe. Rats in the KA-lesioned + exercise group were lesioned with KA and were subjected to swimming exercise for 15 min/d.

### 2.3. Experimental Procedures

An excitotoxic lesion was created in the hippocampus by injecting KA into the lateral ventricles [11]. Rats were first anaesthetized with a cocktail of ketamine (50 mg/mL), xylazine (4.5 mg/mL), and acepromazine (0.4 mg/mL) at a dose of 0.70 mL/kg body weight and were fixed in the stereotaxic apparatus in such a way that the incisor bar was 3.7 mm below the interaural plane. The skull was exposed and a burr hole was drilled using the following coordinates on the right and left sides: anteroposterior 3.7 mm behind the bregma 4.1 mm lateral to the midline [12]. A Hamilton syringe needle filled with kainic acid (0.5 *μ*g/*μ*L) was lowered by 4.5 mm to reach the lateral ventricle and 1.0 *μ*L of KA was injected slowly over a period of 20 min. The needle was withdrawn, skin was sutured, and the animals were kept warm until recovery from anesthesia. Lesioned animals were housed individually. Sham surgery was performed to rule out the effect of surgical injury. Here rats were anaesthetized and fixed in the stereotaxic apparatus and burr hole was drilled as described above. A Hamilton syringe needle was lowered and held in position for 20 min and then withdrawn. The skin was sutured and the animals were returned to their home cages.

### 2.4. Exercise Intervention

Rats were subjected to swimming exercise in a water tank (1.5 m diameter) for 15 min daily for 30 d.

### 2.5. Morphological Procedures

For the cresyl violet staining procedure [11], the animals were deeply anesthetized with ether and fixed on a dissection board and the chest cavity was opened to expose the heart. Fixation following transcardial perfusion was performed using about 15 mL of 0.9% heparinized saline perfused through the left ventricle at 1 mL/min. This was followed by perfusion with 10% formalin, about 250 mL/adult rat, at the same flow rate. The animals were decapitated and 5-6 mm thick coronal sections of brain were removed and kept in 10% formalin for 24 h (following fixation). Paraffin blocks were made by dehydration in 70% alcohol for 2 h and 90% alcohol for 2 h, 3 changes in 100% alcohol for 2 h, clearing with xylene for 2 h, and embedding using 4 changes of paraffin for 0.5 h each, followed by embedding in fresh filtered paraffin. Sections of 5 *μ*m thickness were cut from the middorsal hippocampus and motor cortex regions using a rotary microtome. Sections were selected and mounted serially on air-dried gelatinized slides. The sections were stained with cresyl violet (0.1%) as follows: 100 mg of cresyl violet was dissolved in 100 mL of distilled water. To this, 0.5 mL of 10% acetic acid was added to a pH in the range of 3.5–3.8 log⁡*M*. The stain was filtered before use. For staining, sections were treated with 2 changes of xylene for 10 min, descending grades of alcohol (100%, 90%, and 70%) for 2 min each, distilled water for 15 min, and 0.1% cresyl violet stain for 30 min at 60°C, cooling to room temperature, followed by treatments with ascending grades of 90% and 100% alcohol for 1-2 min each and xylene for 2 min, followed by mounting in DPX.

In each section, CA1, CA3, and dentate gyrus regions of hippocampus, amygdala, and motor cortex regions were selected and the numbers of neurons were counted under light microscopy.

### 2.6. Cell Quantification

Total numbers of survived neurons in the hippocampus (CA1, CA3, and dentate gyrus) and amygdala (basolateral nucleus) were counted. In the case of motor cortex, the number of survived neurons in 10 randomly selected fields, at 40x magnification (Magnus, Olympus Pvt. Ltd. New Delhi, India), was counted and averaged. Cells that were darkly stained and shrunken and fragmented nuclei were excluded from the count. To avoid bias, slides from different groups were coded while counting.

### 2.7. Golgi-Cox Staining

For Golgi-Cox staining, a procedure described by Shankaranarayana Rao and Raju 2004 [13] was followed with some modifications. Briefly, the rats were deeply anesthetized with ether and decapitated. The brains were quickly removed and placed in a Petri dish containing freshly prepared Golgi-Cox fixative. The hippocampus was dissected from both hemispheres of the brain. Motor cortex was dissected out and preserved separately. The caudal half of the other hemisphere with the hippocampus and amygdala region was collected in a bottle for further processing. Tissues collected from individual animals were fixed in individual bottles as follows: brains were kept (as fresh as possible, no perfusion or fixation) on glass wool or gauge in clean bottles and were covered with Golgi-Cox solution and left at room temperature in the dark room. The Golgi-Cox solution was changed after 2 d. Tissues were exposed to the fixative for 2 weeks. After 2 weeks of impregnation in Golgi-Cox solution, the brains were processed further for dehydration in the following order: 50% ethanol for 1 h, 70% ethanol for 1 h, 90% ethanol for 2 h, and 100% ethanol for 1 h. The tissue blocks were then blotted to remove the absolute alcohol from their surface, after which they were carefully mounted on a tissue holder by applying 2 drops of Fevi kwik on the wooden block and the tissue was fixed. Sections were cut using a base sledge microtome. Coronal and horizontal sections of the hippocampus and coronal sections of the amygdala and motor cortex were cut at a thickness of 120 *μ*m. The sections taken were further processed using a soft painting brush in the following order: sections were collected in 70% ethanol and washed in distilled water for 5 min, 5% sodium carbonate for 20 min, distilled water for 5 min, 70% ethanol for 10 min consisting of 2 washes for 5 min each, 90% ethanol for 10 min consisting of 2 washes for 5 min each, 100% ethanol for 10 min consisting of 2 washes for 5 min each, cedar wood oil for 1 h, and xylene for 10 min consisting of 2 washes for 5 min each. Sections were mounted on a glass slide using DPX. Clearing was presumed to be complete once the floating sections in the xylene started sinking.

These sections were observed for their translucency. Translucent sections were mounted serially on a slide using DPX as the mounting media. After the cover slip has been placed, it was “banked up” with excess DPX on all-four sides. Care was taken to avoid inclusion of air bubbles. The slides were air dried horizontally for 1 week. Two days after mounting, ringing was done using DPX to prevent the entry of air bubbles into the slide.

### 2.8. Dendritic Quantification

The dendritic quantification of hippocampal CA1, CA3, basolateral amygdala, and motor cortex neurons was done using the camera lucida technique. From each rat, 8–10 well-stained hippocampal CA1 and CA3 neurons and 8–10 basolateral amygdala and motor cortex neurons were traced using a camera lucida tracing device (Datta Scientifics, Bangalore). Only pyramidal neurons confined to the CA1 and CA3 regions of hippocampus were selected for tracing. All types of neurons from the different nuclei of amygdala and motor cortex were selected for tracing. Neurons that were darkly stained throughout their arborization were selected. Neurons with truncated dendritic branches within a 100 *μ*m radius from the cell body were excluded. Only those neurons that were relatively isolated from neighboring impregnated neurons and neuroglial cells were selected, based on our experience that densely impregnated cells very close to each other interfere with the analysis. The concentric circle method of Sholl (1956) [14] was used for dendritic quantification. On a transparent sheet, concentric circles were drawn. The radial distance between 2 adjacent concentric circles was equivalent to 20 *μ*m. During dendritic quantification, the sheet with concentric circles was placed on the camera lucida-traced neuron in such a way that the approximate center of the cell body of the neuron coincided with the center of the concentric circles. The number of branch points between the 2 successive concentric circles, that is, within each successive 20 *μ*m radial sphere, was counted. The dendritic intersection was defined as the point where a dendrite touches or intersects the concentric circle. The number of dendritic intersections at each concentric circle was counted by placing the transparent sheet with concentric circles on the camera lucida-traced neurons. Both branch points and intersections were counted up to a radial distance of 100 *μ*m from the center of the soma.

### 2.9. Data Analysis

Data was analyzed using two-way analysis of variance (ANOVA) followed by Student's *t*-test (*post hoc*) using GraphPad Prism, version 5.

## 3. Results

**Figure 1:**
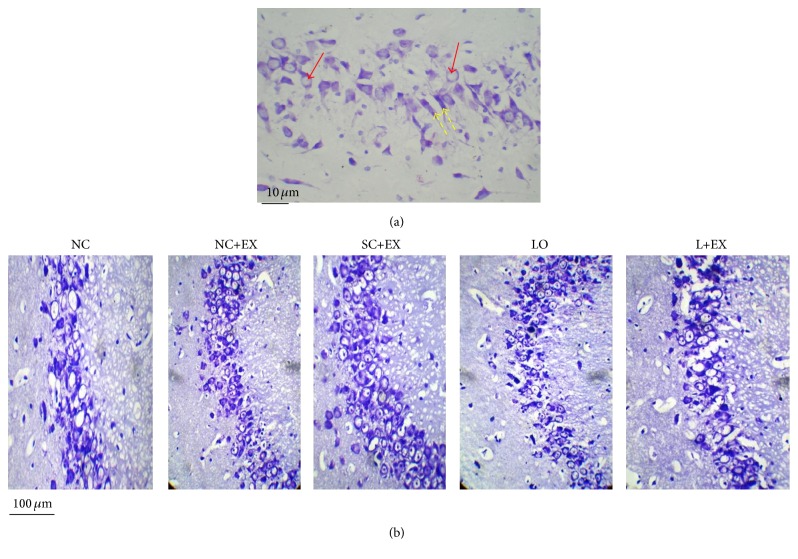
Effects of 30 d swimming exercise on neurons in area CA3 of hippocampus. (a) Photomicrograph of how healthy surviving neurons (solid-line arrow) were differentiated from necrotic neurons (dashed-line arrow) in a small area. (b) Series of photomicrographs showing the surviving neurons in groups of 4-month-old male Wistar rats exposed to the following conditions: normal control (NC), normal control followed by swimming exercise (NC+EX), sham-operated control followed by swimming exercise (SC+EX), and kainic acid-induced lesioning and seizures (LO) followed by immediate, 1 d postlesion exposure to swimming exercise (L+EX) treatments. Magnification 40x.

**Figure 2:**
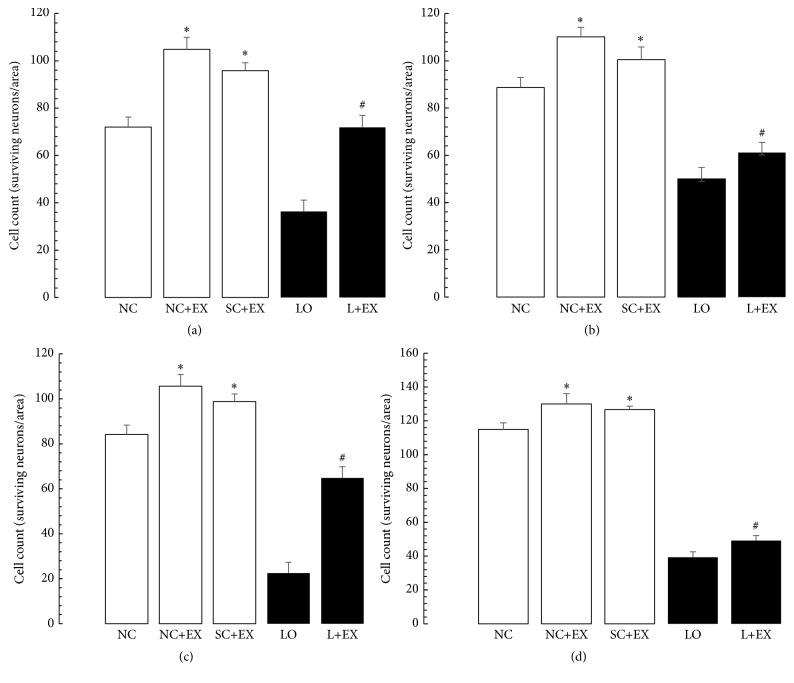
Effects of 30 d swimming exercise on morphometric cell counts in areas CA1 and CA3 of hippocampus. Morphometric cell counts of the surviving neurons in groups of 4-month-old male Wistar rats exposed to the following conditions: normal control (NC), normal control followed by swimming exercise (NC+EX), sham-operated control followed by swimming exercise (SC+EX), and kainic acid-induced lesioning and seizures (LO) followed by swimming exercise (L+EX) treatments. (a and b) Area CA1 of hippocampus. (c and d) Area CA3 of hippocampus. (a and c) Effects of immediate (1 d postgrouping for controls, 1-day postictal period for lesioned rats) exposure to the swimming exercise. (b and d) Effects of delayed (60 d postgrouping for controls, 60 d postictal period for lesioned rats) exposure to the swimming exercise in areas CA1 and CA3, respectively. Bars represent number of surviving neurons expressed in means ± standard errors. Intergroup differences significant at ^**∗**^ *P* < 0.05 and ^#^ *P* < 0.01. *F*-values for (a–d) 402.4, 201.3, 189.3, and 761.1, respectively.

Figure 1(a) demonstrates how we differentiated healthy surviving neurons from necrotic ones, used for counting surviving neurons in this study.  Figure 1(b) shows the effects of the swimming exercise intervention, with and without KA lesioning, on the CA3 area of hippocampus, by light microscopy.  Figure 2 summarizes the effects of the swimming exercise intervention on the morphometric cell counts of surviving neurons in the CA1 and CA3 areas. These data demonstrate that the swimming exercise intervention was associated with significant increases in the numbers of surviving neurons in these hippocampal areas, both in the presence and in the absence of KA lesioning. These increments in surviving neurons were observed in the animals subjected to the exercise intervention 1 d after grouping in the controls, 1 d after KA lesioning in the experimental rats, 60 d after grouping in the controls, and 60 d after KA lesioning in the experimental rats.  Figure 2(a) shows that, in hippocampal area CA1, the cell counts were increased by immediate exposure to the swimming exercise in the normal control, sham-operated control, and KA-lesioned animals.  Figure 2(b) demonstrates similar results associated with delayed exposure to the swimming exercise, albeit a smaller percentage increment in the surviving neuron count after KA lesioning. Figures 2(c) and 2(d) show that a similar pattern was found in hippocampal area CA3.

**Figure 3:**
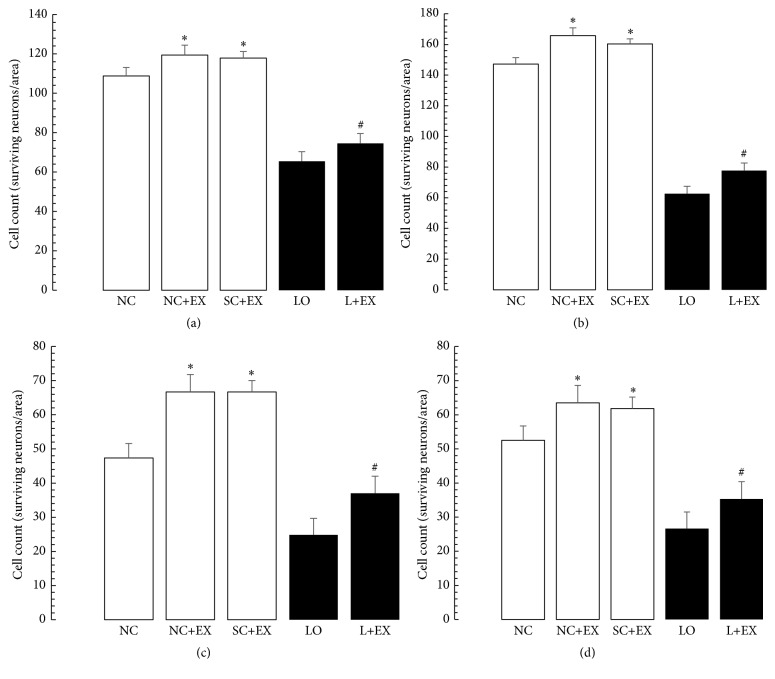
Effects of 30 d swimming exercise on morphometric cell counts in dentate gyrus of hippocampus and basolateral nuclei of amygdala. Morphometric cell counts of the surviving neurons in groups of 4-month-old male Wistar rats exposed to the following conditions: normal control (NC), normal control followed by swimming exercise (NC+EX), sham-operated control followed by swimming exercise (SC+EX), and kainic acid-induced lesioning and seizures (LO) followed by swimming exercise (L+EX) treatments. (a and b) Dentate gyrus of hippocampus. (c and d) Basolateral nuclei of amygdala. (a and c) Effects of immediate (1 d postgrouping for controls, 1 d postictal period for lesioned rats) exposure to the swimming exercise. (b and d) Effects of delayed (60 d postgrouping for controls, 60 d postictal period for lesioned rats) exposure to the swimming exercise in dentate gyrus and basolateral amygdala, respectively. Bars represent number of surviving neurons expressed in means ± standard errors. Intergroup differences significant at ^*∗*^ *P* < 0.05 and ^#^ *P* < 0.01. *F*-values for (a–d) 307.1, 972.2, 112.6, and 173.7, respectively.

**Figure 4:**
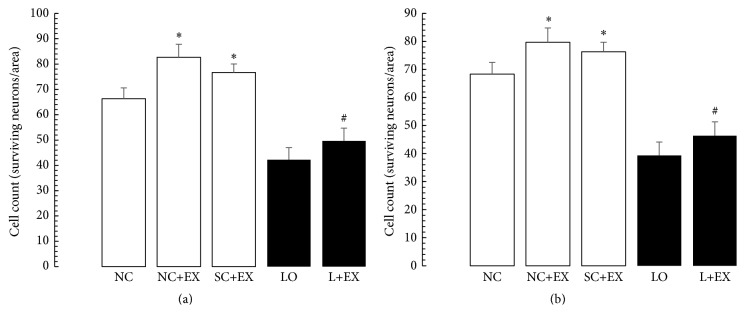
Effects of 30 d swimming exercise on morphometric cell counts in motor cortex. Morphometric cell counts of the surviving neurons in groups of 4-month-old male Wistar rats exposed to the following conditions: normal control (NC), normal control followed by swimming exercise (NC+EX), sham-operated control followed by swimming exercise (SC+EX), and kainic acid-induced lesioning and seizures (LO) followed by swimming exercise (L+EX) treatments. (a) Effects of immediate (1 d postgrouping for controls, 1 d postictal period for lesioned rats) exposure to the swimming exercise. (b) Effects of delayed (60 d postgrouping for controls, 60 d postictal period for lesioned rats) exposure to the swimming exercise. Bars represent number of surviving neurons expressed in means ± standard errors. Intergroup differences significant at ^**∗**^ *P* < 0.05 and ^#^ *P* < 0.01. *F*-values for (a and b) 172.3 and 205.2, respectively.

Figures 3 and 4 summarize the effects of the swimming exercise, with and without KA lesioning, on the surviving neurons in the dentate gyrus, basolateral amygdala, and motor cortex by morphometric counting. These data demonstrate that the swimming exercise also produced significant increases in the numbers of surviving neurons in these areas of the brain, both in the presence and in the absence of KA lesioning. These increments in surviving neurons were observed in the animals subjected to the exercise intervention 1 d after grouping in the controls, 1 d after KA lesioning in the experimental rats, 60 d after grouping in the controls, and 60 d after KA lesioning in the experimental rats.  Figure 3(a) demonstrates that, similar to what was found in areas CA1 and CA3, in dentate gyrus of hippocampus, the cell counts were increased by immediate exposure to the swimming exercise in the normal control, sham-operated control, and KA-lesioned animals.  Figure 3(b) demonstrates similar results associated with delayed exposure to the swimming exercise in the surviving neuron count after KA lesioning. Figures 3(c) and 3(d) show similar findings in basolateral nuclei of amygdala as those in dentate gyrus of hippocampus. Figures 4(a) and 4(b) show similar findings in motor cortex as those in both dentate gyrus and amygdala.

**Figure 5:**
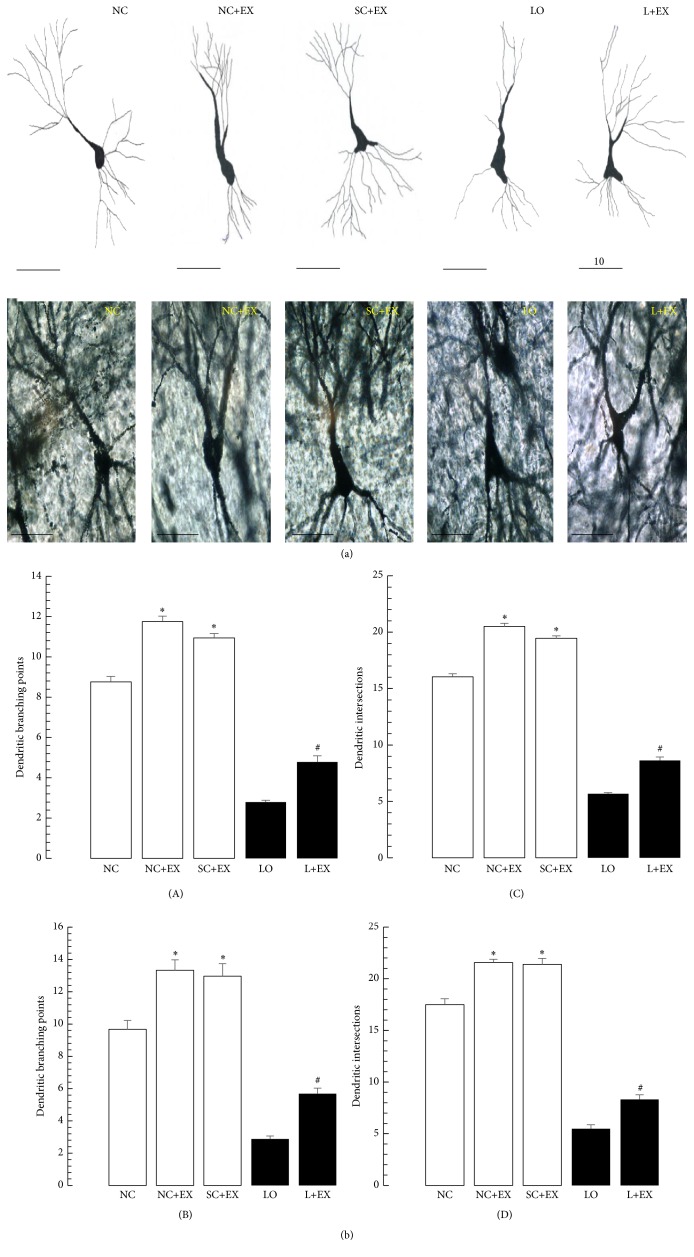
Effects of 30 d swimming exercise on dendritic branch points and intersections in area CA3 of hippocampus. Photomicrographs and histogram bars showing the dendritic branch points and intersections of the surviving neurons in groups of 4-month-old male Wistar rats exposed to the following conditions: normal control (NC), normal control followed by swimming exercise (NC+EX), sham-operated control followed by swimming exercise (SC+EX), and kainic acid-induced lesioning and seizures (LO) followed by immediate, 1 d postlesion exposure to swimming exercise (L+EX) treatments. Magnification 40x. (A) Effects on dendritic branch point counts for immediate (1 d postgrouping for controls, 1 d postictal period for lesioned rats) exposure to the swimming exercise. (B) Effects on dendritic branch point counts for delayed (60 d postgrouping for controls, 60 d postictal period for lesioned rats) exposure to the swimming exercise. (C) Effects on dendritic intersection counts for immediate (1 d postgrouping for controls, 1 d postictal period for lesioned rats) exposure to the swimming exercise. (D) Effects on dendritic intersection counts for delayed (60 d postgrouping for controls, 60 d postictal period for lesioned rats) exposure to the swimming exercise. Bars represent dendritic branch points or intersections in means ± standard errors. Intergroup differences significant at ^**∗**^ *P* < 0.05 and ^#^ *P* < 0.01. *F*-values for (A–D) 479.2, 526.9, 2610, and 1491, respectively.

Figure 5(a) shows the effects of the swimming exercise on the dendritic branch points and intersections of the surviving neurons in the CA3 area of hippocampus by light microscopic camera lucida tracings. Figure 5(b) shows the morphometric counts of dendritic branch points and intersections for the same hippocampal area CA3. Figures 5(b)(A and C) show that, in hippocampal area CA3, the dendritic branch points and intersections, respectively, were increased by immediate exposure to the swimming exercise in the normal control, sham-operated control, and KA-lesioned animals. Figure 5(b)(B) demonstrates similar results associated with delayed exposure to the swimming exercise, albeit a larger percentage increment in the dendritic branch point count of surviving neurons after KA lesioning. Figure 5(b)(D) shows that a similar pattern was found for the dendritic intersection counts in area CA3, without the aforementioned greater percentage increment after delayed exposure to the swimming exercise.

**Figure 6:**
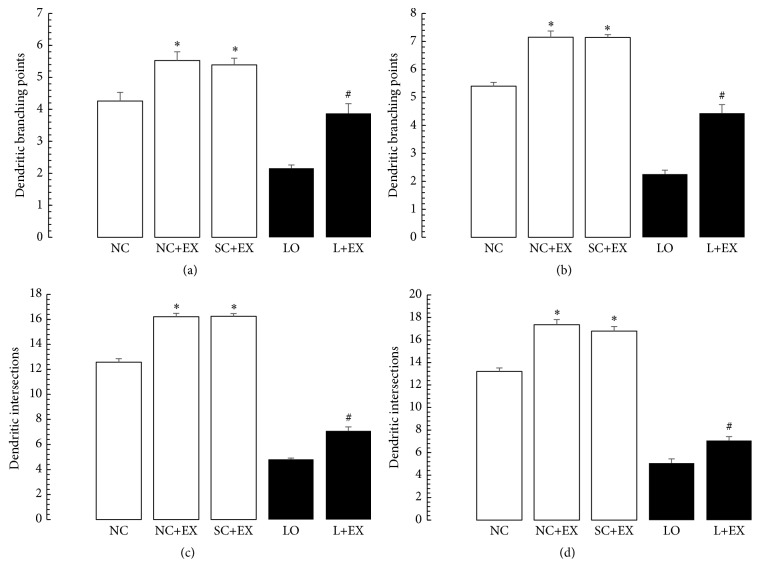
Effects of 30 d swimming exercise on morphometric counts of dendritic branch points and intersections in area CA1 of hippocampus. Morphometric cell counts of dendritic branch points of the surviving neurons in groups of 4-month-old male Wistar rats exposed to the following conditions: normal control (NC), normal control followed by swimming exercise (NC+EX), sham-operated control followed by swimming exercise (SC+EX), and kainic acid-induced lesioning and seizures (LO) followed by swimming exercise (L+EX) treatments. (a) Effects on dendritic branch point counts for immediate (1 d postgrouping for controls, 1 d postictal period for lesioned rats) exposure to the swimming exercise. (b) Effects on dendritic branch point counts for delayed (60 d postgrouping for controls, 60 d postictal period for lesioned rats) exposure to the swimming exercise. (c) Effects on dendritic intersection counts for immediate (1 d postgrouping for controls, 1 d postictal period for lesioned rats) exposure to the swimming exercise. (d) Effects on dendritic intersection counts for delayed (60 d postgrouping for controls, 60 d postictal period for lesioned rats) exposure to the swimming exercise. Bars represent dendritic branch points or intersections in means ± standard errors. Intergroup differences significant at ^*∗*^ *P* < 0.05 and ^#^ *P* < 0.01. *F*-values for (a–d) 182.9, 654.7, 893.6, and 1249, respectively.

Figure 6 summarizes the effects of the swimming exercise on the morphometric counts of branch points and intersection in hippocampal area CA1. These data show that exposure to the swimming exercise was associated with significant increases in the branch points and intersections in these hippocampal areas, in the presence and in the absence of KA lesioning. These increments in branch points and/or intersections of surviving neurons were observed in the animals subjected to the exercise intervention 1 d after grouping in the controls, 1 d after KA lesioning in the experimental rats, 60 d after grouping in the controls, and 60 d after KA lesioning in the experimental rats. Figures 6(a)–6(d) depict a similar pattern in hippocampal area CA1 in regard to the increase in dendritic branch points and intersection associated with the swimming exercise and the KA lesioning as was found in area CA3, with similar percent increments after delayed exposure compared to after immediate exposure to the exercise.

**Figure 7:**
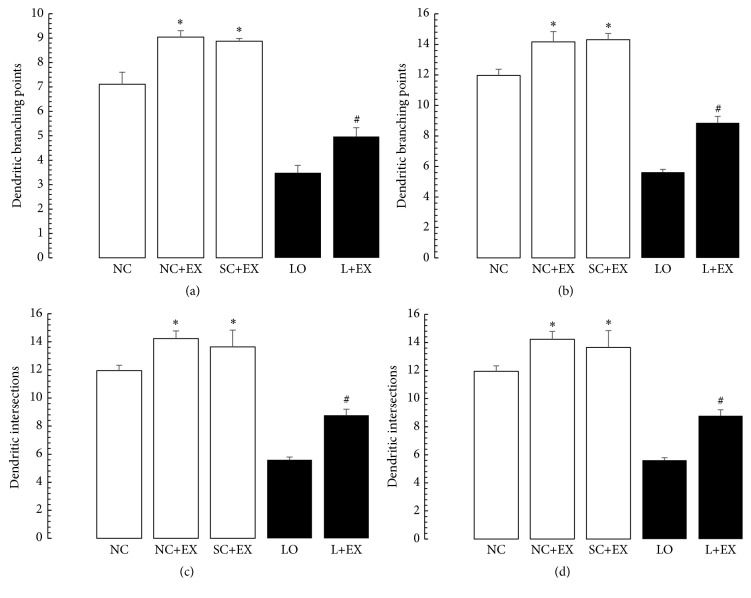
Effects of 30 d swimming exercise on morphometric counts of dendritic branch points in basolateral nuclei of amygdala. Morphometric cell counts of dendritic branch points of the surviving neurons in groups of 4-month-old male Wistar rats exposed to the following conditions: normal control (NC), normal control followed by swimming exercise (NC+EX), sham-operated control followed by swimming exercise (SC+EX), and kainic acid-induced lesioning and seizures (LO) followed by swimming exercise (L+EX) treatments. (a) Effects of immediate (1 d postgrouping for controls, 1 d postictal period for lesioned rats) exposure to the swimming exercise. (b) Effects of delayed (60 d postgrouping for controls, 60 d postictal period for lesioned rats) exposure to the swimming exercise. Bars represent dendritic branching points and intersections in means ± standard errors. Intergroup differences significant at ^*∗*^ *P* < 0.05 and ^#^ *P* < 0.01. *F*-values for (a and b) 317.7 and 182.9, respectively.

**Figure 8:**
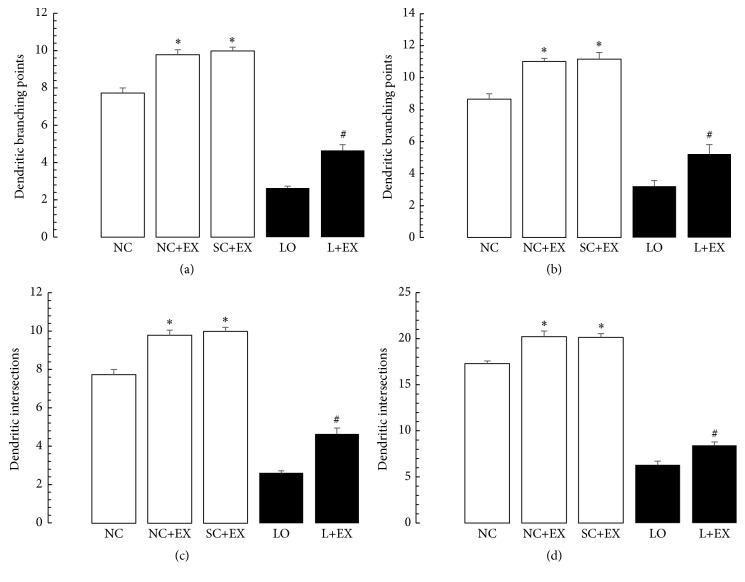
Effects of 30 d swimming exercise on morphometric counts of dendritic branch points and intersections in motor cortex. Morphometric cell counts of dendritic branch points and intersections of the surviving neurons in groups of 4-month-old male Wistar rats exposed to the following conditions: normal control (NC), normal control followed by swimming exercise (NC+EX), sham-operated control followed by swimming exercise (SC+EX), and kainic acid-induced lesioning and seizures (LO) followed by swimming exercise (L+EX) treatments. (a) Effects on dendritic branch point counts for immediate (1 d postgrouping for controls, 1 d postictal period for lesioned rats) exposure to the swimming exercise. (b) Effects on dendritic branch point counts for delayed (60 d postgrouping for controls, 60 d postictal period for lesioned rats) exposure to the swimming exercise. (c) Effects on dendritic intersection counts for immediate (1 d postgrouping for controls, 1 d postictal period for lesioned rats) exposure to the swimming exercise. (d) Effects on dendritic intersection counts for delayed (60 d postgrouping for controls, 60 d postictal period for lesioned rats) exposure to the swimming exercise. Bars represent dendritic branching points or intersections in means ± standard errors. Intergroup differences significant at ^*∗*^ *P* < 0.05 and ^#^ *P* < 0.01. *F*-values for (a–d) 324.9, 594, 1000, and 1421, respectively.

Figure 7 shows the effects of the exercise intervention on the dendritic branch points and intersections of the surviving neurons in a basolateral amygdala.  Figure 8 presents the effects of the swimming exercise on the morphometric counts of branch points and intersections in motor cortex. These data demonstrate that the swimming exercise also increased the dendritic arborization in these areas of the rat brain, in the presence and in the absence of KA lesioning. These increments in branch points and/or intersections of surviving neurons were observed in the animals subjected to the exercise intervention 1 d after grouping in the controls, 1 d after KA lesioning in the experimental rats, 60 d after grouping in the controls, and 60 d after KA lesioning in the experimental rats. Figures 7(a)–7(d) and 8(a)–8(d) show virtually the same pattern of change with respect to the swimming exercise, in the presence and absence of KA lesioning, in basolateral nuclei of amygdala and motor cortex, respectively, as was found in area CA3 of hippocampus.

These findings suggest that delayed exposure to the swimming exercise may be associated with approximately the same percentage increase in counts of surviving neurons after KA lesioning in dentate gyrus, basolateral nuclei of amygdala, and motor cortex. Compared to these areas, there appear to be a relatively smaller percentage increase in surviving cell counts in hippocampal areas CA1 and CA3 and a greater percentage increment in dendritic branch points in area CA3 with delayed exposure to the exercise.

## 4. Discussion

The main finding of this study is that aerobic (swimming) exercise increases neurogenesis in five discrete areas of the brain following chemically induced (kainate) seizures in a rat model for temporal lobe epilepsy. This exercise-induced neurogenesis occurred whether the exercise treatment was initiated in the 1 d or 60 d postictal period. This finding is bolstered by our control studies in which the same swimming exercise increased neurogenesis in normal control animals and in sham-operated control animals that were subjected to the same swimming exercise regimen.

This is the first study to determine the effects of swimming exercise on neurogenesis in hippocampal areas CA1, CA3, and dentate, amygdala, and motor cortex in the same animals. We defined neurogenesis as an improvement in morphometric counts of surviving neurons and of their dendritic branch points and intersections, evaluated in three areas of the hippocampus, in the amygdala, and in the motor cortex. The exercise treatment increased the total number of neurons of all the five brain areas studied in both the normal and the sham-operated control rats. Swimming exercise is known to be an effective stimulus to neurogenesis and neurogenesis has been positively correlated with improved cognitive functions in both experimental animals and humans [8]. Neurogenesis has mostly been studied in the neural progenitor (stem) cells within the subgranular zone of the hippocampal dentate gyrus and subventricular zone of the olfactory bulb in adult rats [15].

Neurogenesis is also known to occur in areas CA1 and CA3 of hippocampus, in amygdala [3], and in sensorimotor cortex [16] but motor cortex neurogenesis has not been extensively studied. Nerve growth factor (NGF) is the central nervous system's protection against excitotoxicity [17]. In neural progenitor stem cells, NGF is transported by axons via retrograde transport to cell bodies where it stimulates the growth and differentiation of the progenitor cells [18]. NGF is shown to bind a member of the tyrosine kinase receptor family known as tropomyosin receptor kinase A (TrkA) with high affinity [19] and also to the p75 low-affinity nerve growth factor receptor (LNGFR) [20]. NGF, together with brain-derived neurotrophic factor (BDNF), stimulate neural stem cells, also after binding to LNGFR as well as to TrkB [21–23]. TrkB is shown to be a regulator of neural plasticity and is upregulated following scavengers protecting neurons from oxidative stress, thereby promoting neural tissue regeneration [24]. Simvastatin, niacin, and low-level laser therapies are known to upregulate both TrkB and BDNF and are reported to increase axonal and neurite growth in rats subjected to ischemic stroke by occlusion of the middle cerebral artery [25–27]. BDNF also upregulates neuronal dendrite growth and production of postsynaptic density protein-95, thereby reorganizing synapses (synaptogenesis) and promoting neural plasticity [28]. It is well established that regular exercise can enhance brain functions, including cognitive abilities, and upregulate neurotrophins [29]. Upregulation of neurotrophins has been widely reported following swimming exercise in rats, correlated with improved brain functions [30].

### 4.1. Exercise and Neurogenesis in Normal and Sham-Operated Control Rats

We measured neurogenesis in hippocampal dentate gyrus and areas CA1 and CA3, as well as in amygdala and motor cortex in separate groups of control animals at two time points—immediately after grouping or sham operation and 60 days after grouping or sham operation. It was beyond the scope of this study to determine the precise mechanism of neurogenesis but, in accordance with previous research cited above, it is likely that the swimming exercise increased the production of neurotrophic factors in the brain areas studied. Our results on the morphometric counting of neuronal cell bodies and of dendritic branch points and intersections exhibited similar patterns in each of the aforementioned brain areas. These findings in our normal control and sham-operated rats demonstrate that total numbers of neurons and dendritic branch points and intersections were increased significantly by the swimming exercise whether or not the exercise treatment was administered immediately or after a delay of 60 d following grouping or sham surgery. The purpose of studying this delay was to provide control data for a second experiment in which we evaluated the effects of the same intensity and duration of swimming exercise initiated in the immediate 1 d and delayed 60 d postictal period on kainate-treated rats of the same strain, age, and sex as the controls.

### 4.2. Exercise and Neurogenesis in Kainate-Treated Rats

We compared neurogenesis after administering kainic acid and observing seizures with and without the same 30 d swimming exercise treatment in the same brain areas of the hippocampus, amygdala, and motor cortex as in experiment 1 on the normal and sham-operated control rats. Effects of the swimming exercise treatments were also compared for groups of rats subjected to the swimming exercise regimen in the immediate 1 d and delayed 60 d postictal periods. Kainate-induced seizures in mice are reported to preferentially activate a specific subpopulation of cells, glial-like radial neural stem cells [31, 32], distinct from the subpopulation of neural stem cells stimulated by running exercise [33]. It is thought that such stem cells have the capacity to migrate throughout the brain [34]. We did not identify the subtype or the migration pattern of the neural stem cells stimulated by kainate-induced lesioning and seizures [35, 36]. Our morphometric counting of cell bodies and dendritic branch points and intersections provided a measure of the number of neurons surviving the kainate lesioning and the number of dendritic arborizations available for synaptogenesis [37]. The swimming exercise treatment resulted in significant increases in the number of surviving neurons and dendritic branch points and intersections in all five brain regions and at the two time points studied. These findings suggest that, following kainate lesioning and seizures, there is a significant increase in neurogenesis whether the exercise is initiated and completed within the first 30 postictal days or after a delay of 60 postictal days. This finding is significant because although aerobic exercise is known to augment neurogenesis in both rats and humans, humans affected by temporal lobe epilepsy may be unable to maintain regular exercise regimens, without breaks, over the long term.

### 4.3. Limitations of the Study

One limitation of this study is that we did not mark and differentiate the neural stem cells and mature neurons in each of the brain areas studied. We used cresyl violet staining to histochemically label the Nissl substance, permitting us to count the numbers of surviving neurons and dendritic branch points and intersections. We demonstrated repopulation of the selected brain areas with mature neurons but could not determine the number of neural stem cells present or the efficiency of their transformation into neurons. An immunohistochemical marker for glial-like radial neural stem cells such as glial fibrillary acidic protein in conjunction with neuron-specific enolase [38], beta tubulin, and/or nestin [39] to mark the neurons could permit a determination of the efficiency of neurogenesis, likely to be a more sensitive indicator of recovery than the counting of the surviving neurons marked with cresyl violet. Therefore, absence of specific markers for neurogenesis should be considered as a limitation of this study. However, cresyl violet staining is an acceptable, widely used method for quantifying the changes in neuronal morphology associated with insults to the brain parenchyma, such as ionizing radiation [40] and chemical poisonings [41], including the kainate lesioning model of temporal lobe epilepsy used in the present study.

Another limitation is that we were not able to perform morphometric counting of surviving neurons and dendritic branch points and intersections in the brains of kainate-lesioned animals that were observed for 60 postictal days in the absence of the swimming exercise intervention. This evaluation would have provided a measure of the effectiveness of spontaneous neurogenesis expected, by previously reported research, to be negligible. Kainate-induced seizures are thought to be an effective stimulus for neurogenesis but an excitotoxic “limbic syndrome” is reported to occur at the sites of neurogenesis where damaged neurons may continue to release substances which contribute to kindling of epileptic discharges, evidenced by the presence of mossy fibers expressing kainate receptors, resulting in aberrant glutamatergic synapses [42–44]. This process is thought to contribute to a vicious cycle of seizures commonly observed in patients diagnosed with temporal lobe epilepsy. We observed that when individual kainate-lesioned rats were not housed in separate cages, that is, when they were housed communally, they had a tendency to cannibalize each other. This behavior may be indicative of serious emotional dysregulation that is reported in patients diagnosed with temporal lobe epilepsy patients who are resistant to treatment with antiepileptic drugs [45].

The results of this study must be interpreted cautiously pending confirmation of functional significance by behavioral studies, using the same or similar experimental paradigm. Pyramidal neurons are the most functionally important neurons in the mammalian cerebral cortex. Pyramidal neurons possess both apical and basal dendritic trees with extensive domains giving rise to a multitude of synapses, generating both action potentials and brain waves. These properties provide for highly specialized, parallel information processing mechanisms, thought to be necessary for neural integration and plasticity [46]. However, the amount of dendritic branching may not be indicative of the amount of integration and degree of developmental specialization. Pyramidal cell branching in prefrontal cortex is shown to be greater at birth than in adulthood in macaques [47]. Some cytoarchitectural features are common to all cortical areas and species [48]. Interindividual and interspecies variation in the number of dendritic spines within the dendritic trees may reflect different pyramidal cell phenotypes, patterns of cortical connectivity, and, therefore, functional networking [49]. In sensorimotor cortex of the macaque, variations in dendritic arbor size, number of branch points, spine density, and soma size seem not to be linked suggesting that formation of the networks may be based on functionality rather than on anatomical or genetic programming [50].

## 5. Conclusion

Despite the aforementioned limitations, the main results of this study demonstrate that swimming exercise increases the numbers of surviving neurons as well as the number of dendritic branch points and intersections and intersections in five discrete areas of the rat brain in normal control and sham-operated control rats, as well as in rats subjected to kainate-induced seizures, whether or not there is a 60 d delay in initiating the exercise treatment. Neurogenesis in the rats subjected to the 60 d delay was found to be significantly less than that in the animals that were given the exercise treatment immediately, beginning 1 d after the kainate lesioning and seizures. These findings suggest that aerobic exercise may improve neural plasticity in areas of the brain involved with regulation of emotion, learning, memory, and motor coordination in both normal and epileptic humans by increasing neurogenesis and synaptogenesis; however, such exercise may improve recovery from epileptic seizures even after a substantial delay. Future studies should take into consideration that current clinical guidelines on medical management of temporal lobe epilepsy do not include aerobic exercise as an adjunct to antiepileptic drug treatments.
